# Efficacy and Safety of Epidermal Radionuclide Therapy with ^188^Re Resin in Patients with Nonmelanoma Skin Cancers: Long-Term Data from a Single Center

**DOI:** 10.2967/jnumed.125.270345

**Published:** 2026-02

**Authors:** Paolo Castellucci, Carlotta Baraldi, Luigia Vetrone, Federico Zagni, Claudio Malizia, Alessio Giuseppe Morganti, Lidia Strigari, Emi Dika, Stefano Fanti

**Affiliations:** 1 Nuclear Medicine IRCCS Azienda Ospedaliero-Universitaria S.Orsola, Bologna, Italy;; 2 Oncologic Dermatology Unit, IRCCS Azienda Ospedaliero-Universitaria S.Orsola, Bologna, Italy;; 3 Department of Medical Physics, IRCCS Azienda Ospedaliero-Universitaria di Bologna, Bologna, Italy;; 4 Radiation Oncology IRCCS Azienda Ospedaliero-Universitaria S.Orsola, Bologna, Italy; and; 5 Department of Medical and Surgical Sciences (DIMEC), Alma Mater Studiorum, University of Bologna, Bologna, Italy

**Keywords:** nonmelanoma skin cancer, epidermal radionuclide therapy, ^188^Re, not sealed sources

## Abstract

Nonmelanoma skin cancer (NMSC) occurs in photoexposed areas and is frequent in the elderly population. Surgical excision remains the treatment of choice whenever feasible. However, alternative approaches are clinically important for patients who are unsuitable for surgery. This study evaluated the efficacy and safety of ^188^Re epidermal radionuclide therapy (^188^Re-ERT) for local disease control in NMSCs deemed inoperable. **Methods:** In total, 124 consecutive patients (79 men, 45 women; mean age, 81 y [range, 49–94 y]) with histologic confirmation of NMSC were treated with ^188^Re-ERT, with 181 lesions treated. Efficacy was evaluated 6 mo after ^188^Re-ERT with videodermoscopy and biopsy. Follow-up was performed at 12, 18, 24, and 36 mo. Relapses were confirmed histologically. Safety was assessed on the basis of CTCAE version 5.0. **Results:** Lesion characteristics included surface area (mean, 6.0 cm^2^; range, 1–34 cm^2^), thickness (mean, 1.2 mm; range, 0.2–3.0 mm), volume (mean, 0.85 cm³; range, 0.05–7.7 cm³). Locations include the H area (102 lesions), scalp (39 lesions), extremities (24 lesions), and thorax (16 lesions). Histology confirmed 104 basal cell carcinoma; 71 squamous cell carcinoma, and 6 basosquamous carcinoma. Mean administered dose to the lesion was 51 Gy, the mean dose to the deepest point of cancer invasion was 25 Gy, and the mean treatment duration was 90 min. No patient reported pain during or after the treatment. Overall, 165 of 181 lesions (91.1%) were relapse-free at 36 mo. Relapses occurred in 16 patients as follows: 6 at 6 mo, 3 at 12 mo, 2 at 18 mo, 3 at 24 mo, and 2 at 36 mo. Lesions less than 8 cm^2^ demonstrated a relapse rate of 1.4% at 6 mo. Larger lesion area volumes were significantly associated with relapse. Early adverse skin reactions classified according to CTCAE version as G1 (minimal skin changes, faint erythema, or dry desquamation) or G2 (moderate erythema, patchy moist desquamation, and edema) were observed in 165 of 181 lesions (91.1%) and resolved within a mean time of 32 d; G3 skin reactions (severe skin changes without pain) were present in 16 lesions, lasting up to 8–12 wk. **Conclusion:** ^188^Re-ERT showed high efficacy with a relapse-free rate of 91.1% at 36 mo. Larger lesions were associated with an increased risk of relapse. Adverse events were generally mild, resolving within 32 d. These results support ^188^Re-ERT as a safe and effective alternative for patients with inoperable NMSC.

## Visual Abstract

**Figure:**
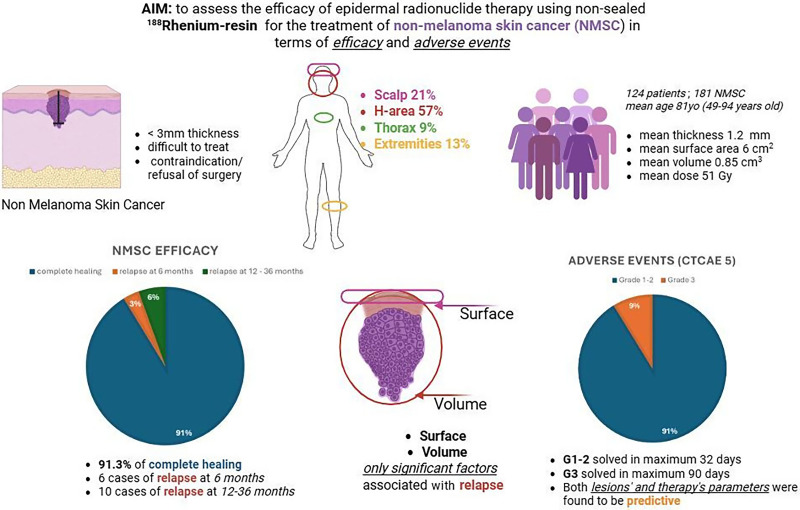

Skin cancer is the most prevalent malignancy worldwide. Approximately 90% of skin cancers are classified as nonmelanoma skin cancers (NMSC), of which about 70% are basal cell carcinoma (BCC) and 20% are squamous cell carcinoma (SCC) (1).

Most NMSCs develop in sun-exposed areas, particularly in the so-called *H area*, which includes the forehead, nose, ears, and cheeks. Other risk factors for NMSCs include having a fair skin phototype, chronic sun exposure, older age, immunosuppression, and human papilloma virus infection (2,3). Mohs micrographic surgery is considered the optimal treatment for primary NMSC with a 5-y cure rate of approximately 95% (4,5). However, surgery might result in poor cosmetic outcomes or prove technically difficult in patients with large lesions localized in areas such as nose wings, ears, eyelids, lips, external genitals, or fingers (5). In such patients, alternative approaches could be considered (6). Alternative treatments should be considered individually, taking into account patient fitness, comorbidities, life expectancy, and potential procedural complications. These include photodynamic therapy, cryotherapy, intralesional 5-fluorouracil, imiquimod 5%, and laser- (7–9) or image-guided superficial radiation therapy. External beam radiation therapy, either with electron beam radiation or with isotope-based or electronic brachytherapy, could also be considered appropriate (10–14). In more advanced stages of disease, immunotherapy and Hedgehog inhibitors also demonstrated promising results, albeit only in selected cases (15–18).

Despite the limited diffusion of ^188^Re epidermal radionuclide therapy (^188^Re-ERT), few reports are promising in terms of efficacy tolerability, low rates of adverse events, and good outcomes (19–23).

Herein, we describe our clinical experience reporting data after several years of ^188^Re-ERT use in our clinical practice. Our main goal was to determine the clinical efficacy of ^188^Re-ERT, and the secondary objective was to evaluate the associated adverse events.

## MATERIALS AND METHODS

The study was performed according to the Declaration of Helsinki; patients provided written informed consent to participate, and the study was approved by the local ethics committee (23/2019/Oss/AOUBo). Between 2019 and 2024, 124 consecutive patients affected by NMSC were enrolled by the Dermatology Unit and treated with ^188^Re-ERT at the Nuclear Medicine Unit of the IRCCS Azienda Ospedaliero-Universitaria, S.Orsola Bologna Italy. ^188^Re is a high-energy radioisotope, emitting 85% β^−^ (2.2 MeV) and 15% γ (155 keV) radiation. ^188^Re releases 92% of its energy within 2 mm of skin depth and almost all its energy within 3 mm (24).

Inclusion criteria included the following: histologically proven cutaneous NMSC, lesion thickness invasion below 3.0 mm according to single or multiple biopsies, lesions categorized as difficult to treat with surgery by an expert dermatologist team according to the criteria proposed by Peris (6), general contraindication for surgery, and refusal of surgery.

According to these criteria, we retrospectively enrolled 124 consecutive patients (45 women, 79 men; mean age, 81 y [range, 49–94 y]), demonstrating 181 histologically proven NMSCs (104 BCC, 71 SCC, and 6 basosquamous cell carcinoma [BSC]).

### Therapy Procedure

Margins of the gross tumor volume (GTV) were drowned by a dermatologist, according to videodermoscopy and biopsy. GTV included the margins of the lesions to treat, plus 2–4 mm safe margins, varying according to size and location of the lesions. Two experienced nuclear medicine specialists used a dedicated device (Rhenium-SCT Skin Cancer Therapy; Oncobeta GmbH) provided with a brush and a carpule filled with highly concentrated ^188^Re resin to apply the resin over the lesion’s margins. Before this, a 7-μm foil was placed over the skin to avoid any direct contact of the resin with the skin. Delivered doses in different points of the lesion’s thickness were measured and reported using 2 independent methods: Varskin 5 software (25) and an in-house tool, proposed by Zagni et al. (26) for predicting the dose distribution based on Monte Carlo code Fluka (27).

The mean dose to the whole lesion volume was 51 Gy, and the mean superficial dose (at 0.001-mm depth) was 165 Gy. The mean dose at 0.50-mm depth was 51 Gy, and the mean target dose to the deepest point of invasion was 25 Gy. The mean treatment time was 90 min (range, 21–285 min). Patients were evaluated after 21–35 d and then 90 and 180 d after ^188^Re-ERT when videodermoscopy was performed followed by a biopsy in the case of suggestive findings. Patients were then checked after 12, 18, 24, and 36 mo. Of the 124 patients, 32 died for other causes during follow-up; the mean follow-up was 36 mo (range, 6–72 mo).

Early adverse events were assessed according to Common Terminology Criteria for Adverse Events (CTCAE) version 5 (28) in the window period of 21–90 d or up to healing of the wound. Definition of adverse events was divided as follows: grade 1 was minimal skin changes such as faint erythema or dry desquamation; grade 2 was moderate erythema, patchy moist desquamation, and edema; grade 3 was severe skin changes, but it is worth emphasizing that even lesions classified as grade 3 did not cause any significant pain.

All statistical analyses were performed using R software (version 4.1.2). A *P* value of less than 0.05 was considered statistically significant. Continuous variables are presented as means or medians based on distribution. Categoric variables are presented as frequencies. The primary endpoint was ^188^Re-ERT efficacy, defined as no relapse at follow-up. Descriptive statistics summarized patient demographics and lesion characteristics: age, sex, lesion type (BCC, SCC, BSC), location, surface area, thickness, and volume.

Comparisons between different lesion types (BCC, SCC, BSC) and locations (H area, scalp, extremities, trunk) were performed using the Mann–Whitney *U* test for continuous variables and the χ^2^ test or Fisher exact test for categoric variables. Univariate logistic regression models were used to identify predictors of relapse (Supplemental Table 1; supplemental materials are available at http://jnm.snmjournals.org) and CTCAE (Supplemental Table 2) and included age, sex, lesion type, lesion location, surface area, thickness, volume, and doses received at different depths. Odds ratios (ORs) and 95% CIs were reported.

## RESULTS

**FIGURE 1.**
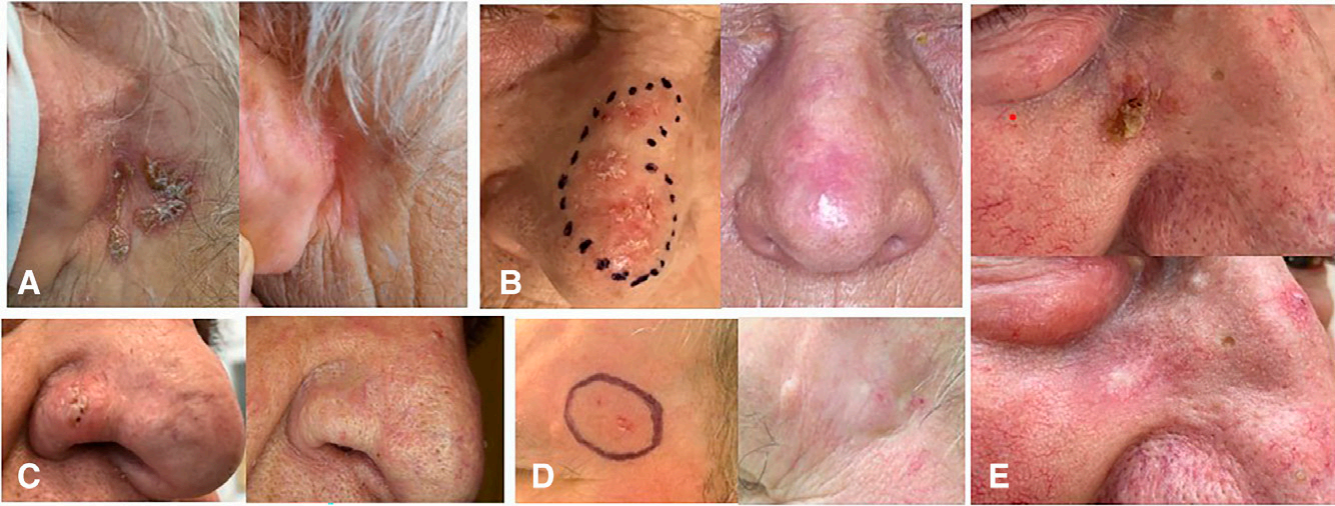
Series of small lesions in typical common locations in H area, before and 2 y after ^188^Re-ERT. (A) SCC retroauricular (area, 7.2 cm^2^; thickness, 1.0 mm); (B) nose SCC (area, 5.6 cm^2^; thickness, 1.7 mm); (C) BCC nodular subtype in right nose wing (area, 2.4 cm^2^; thickness, 2.0 mm); (D) SCC of left temple (area, 6.1 cm^2^; thickness, 1.5 mm); (E) SCC right cheek (area, 2 cm^2^; thickness, 0.8 mm).

Of the 181 treated lesions, 108 (60%) were newly diagnosed, whereas 73 (40%) were relapses after 1 or multiple treatments including photodynamic therapy, cryotherapy, laser or imiquimod (37 lesions), surgery (22 lesions), radiotherapy (14 lesions) including ^188^Re-ERT (3 lesions). The mean surface areas was 6.0 cm^2^ (range, 1–36 cm^2^), the mean thickness was 1.28 mm (range, 0.2–3.0 mm), and the mean volume was 0.85 cm^3^ (range, 0.05–7.7 cm^3^). Lesions were located in the H area (*n* = 102), the scalp (*n* = 39), extremities (*n* = 24), and trunk (*n* = 16). Patients and lesion characteristics are reported in Table 1, with some examples shown in Figure 1 and Supplemental Figure 1.

**TABLE 1.** Patient and Lesion Characteristics

Characteristic	Value
Patient	124
Age (y)	81 (range, 49–94)
Male	79
Female	45
Lesion total	181
BCC	104
Nodular	79
Morphoeic	14
Superficial	11
SCC	71
BSC	5
BSC (BCC-morphoeic BCC and SCC)	1
Mean surface area (cm^2^)	6.0 (range, 1–36)
Mean thickness (mm)	1.2 (range, 0.2–3.0)
Mean volume (cm^3^)	0.85 (range, 0.05–7.7)
Previous treatment	
Newly diagnosed	108
Relapses after following previous treatments	73
Photodynamic, cryo, laser therapy, or imiquimod	37
Surgery	22
RT	11
^188^Re	3
Location	
H area	102
Scalp	39
Extremities	24
Trunk	16

Qualitative data are number, unless otherwise indicated.

During follow-up, we observed relapses in 16 of 181 lesions (efficacy rate, 91.1%). However, 5 of 16 relapses were at the margins of the treated area (GTV_edge_) and partially not covered by ^188^Re-ERT, whereas 11 of 16 relapses were fully in the treated area. Relapses were assessed by videodermoscopy and all confirmed by biopsy; 6 lesions relapsed at the 6-mo checkpoint (2 in GTV_edge_), 3 lesions after 12 mo, 2 lesions after 18 mo (1 in GTV_edge_), 3 lesions after 24 mo (1 in GTV_edge_), and 2 lesions after 36 mo (1 in GTV_edge_). Efficacy results are summarized in Table 2. Relapsed lesions were subsequently treated with other treatments: 8 lesions with surgery or microsurgery, 3 with ^188^Re-ERT (1 is scheduled for retreatment); 3 lesions were treated with imiquimod, cryotherapy, or a combination of the two.

**TABLE 2.** Efficacy Results*

Relapse rate	Location of the relapse	Location of the lesion	Histology	Mean surface area (cm^2^)	Mean thickness (mm)
After 6 mo	4 GTV	2 H	1 BCCnod	9	1
		2 Ex	1 BCC-Morph		
			2 SCC		
	2 GTV_edge_	1 T	1 BCCnod	22	2
		1 Sc	1 SCC		
After 12 mo	3 GTV	^1^H	2 BCC-Morph	4	1.5
		1 T	1 SCC		
		1 Ex			
After 18 mo	1 GTV	1 Sc	1 BCCnod	15	0.8
	1 GTV_edge_	1 Sc	1 BCCnod	25	1.5
After 24 mo	2 GTV	^1^H	1 BCCnod	9.5	1.1
		1 Ex	1 SCC		
	1 GTV_edge_	^1^H	1 BCCnod	19	1.1
After 36 mo	1 GTV	1 Sc	1 SCC	11.5	0.4
	1 GTV_edge_	1 Sc	1 SCC	4	1.2
All relapsed lesions	16	5/102 H	7/71 SCC	13.3	1.1
		5/39 Sc	6/79 BCCnod		
		4/24 Ex	3/15 BCC-Morph		
		2/16 T			

* Characteristics of relapsed lesions during follow-up are reported.

H = H area; Sc = scalp; Ex = extremities; T = thorax; BCCnod = nodular subtype BCC; BCC-Morph = morphoeic subtype.

We observed a strict correlation between lesion size (expressed as surface area and volume) and the efficacy rate (Table 2). Using an arbitrary cutoff of 8 cm^2^ in surface area, we observed only 2 relapses out of 139 treated lesions after a 6-mo follow-up (efficacy rate, 98.6%) and 6 of 137 more relapses after a mean follow-up of 36 mo (efficacy rate, 95.6%).

**FIGURE 2.**
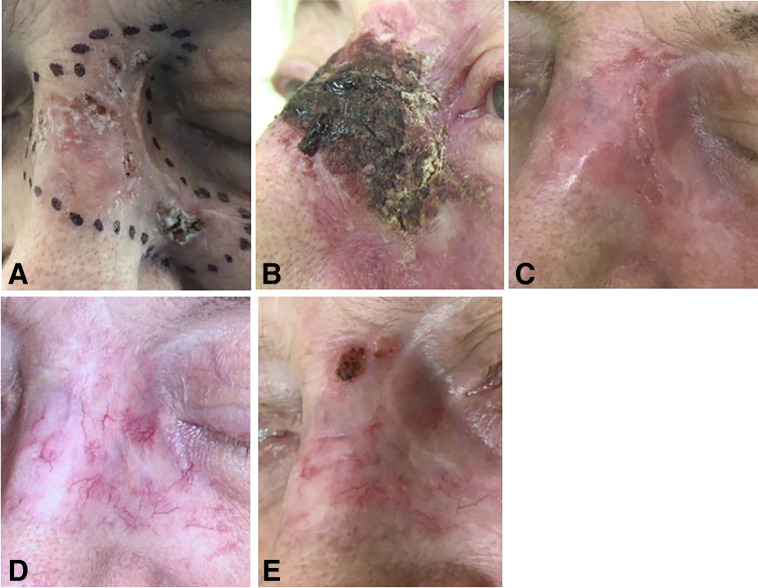
86-y-old woman with relapse after surgery of morphoeic BCC, involving nose, left cheek, and partial left eyelid. Lesion area, 10 cm^2^; thickness, 0.6 mm; administered dose, 430 MBq of ^188^Re resin; mean absorbed dose, 54 Gy; treatment time, 61 min. (A) Before treatment: visible scar from previous surgery. (B) At 30 d after ^188^Re-ERT (CTCAE grade 3). (C) At 60 d after ^188^Re-ERT. (D) At 120 days after ^188^Re-ERT: complete resolution of wound, with multiple telangiectasia and depigmentation. (E) At 180 days after ^188^Re-ERT: evidence of small relapse, subsequently treated with microinvasive surgery. Patient died of unrelated causes 2 y after ^188^Re-ERT.

**FIGURE 3.**
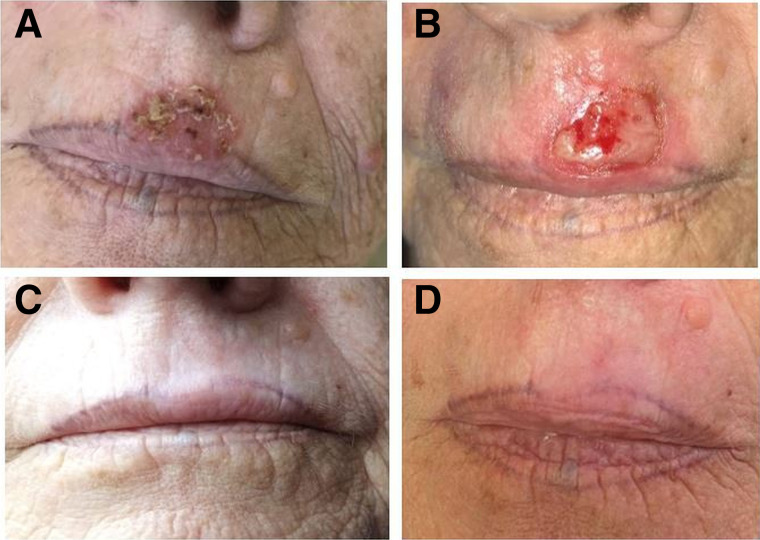
78-y-old woman with relapse of SCC of upper lip, previously treated with cryotherapy. Lesion area, 4 cm^2^; thickness, 1.7 mm; administered dose, 190 MBq of ^188^Re resin; mean absorbed dose, 60 Gy; treatment time, 110 min. (A) Before treatment. (B) At 28 d after ^188^Re-ERT (CTCAE grade 3). (C) At 180 days after ^188^Re-ERT. (D) At 2 y after ^188^Re-ERT, faint depigmentation visible.

In 165 of 181 lesions, early skin adverse events resolved within 4 wk after the first visit and were classified as grades 1–2 according to CTCAE version 5 (28). In the remaining 16 lesions, we observed a grade 3 (CTCAE version 5) lasting up to 8–12 wk but all (Figs. 2 and 3; supplemental materials), except 2, resolved within 90 d. Early after treatment, 98 of 181 lesions needed support to heal the wound, mostly with the application of ialuronic acid, antibiotic creams, steroids, or combinations. Safety results are reported in Table 3.

**TABLE 3.** Adverse Events Results*

CTCAE grade	No. lesions	Age (y)	Mean surface area (cm^2^)	Mean thickness (mm)	Lesion volume (cm^3^)	Location	Mean duration to heal (wk)	Mean dose received (Gy)	Local topic medical support
1	79	80	5.5	1.0	0.6	46 H	2.8	48	28/79
						20 Sc			
						10 Ex			
						3 T			
2	86	81	5.8	1.3	0.8	50 H	4	50	38/86
						18 Sc			
						9 Ex			
						9 T			
3	16	78	9.5	1.6	1.5	6 H	9	65	16/16
						5 Ex			
						4 T			
						1 Sc			

* Characteristics of relapsed lesions during follow-up are reported.

H = H area; Sc = scalp; Ex = extremities; T = thorax.

Univariate logistic regression analysis shows that lesion area is a significant predictor of relapse at 6 mo (OR, 1.11; *P* = 0.01) and 18 mo (OR, 1.16; *P* = 0.02) (supplemental materials), suggesting its value in early and intermediate-term relapse prediction. However, its predictive power diminishes at 12, 24, and 36 mo. Additionally, early adverse event severity (grades 1 and 2 vs. grade 3) is associated with lesion thickness (OR, 2.48; *P* = 0.03), area (OR, 1.07; *P* = 0.03), volume (OR, 1.48; *P* = 0.01), and various therapy parameters, including administered dose (OR, 1.004; *P* = 0.01) and depth-specific doses (*P* = 0.01 for all; supplemental materials).

## DISCUSSION

The clinical use of ^188^Re-ERT for NMSC was first reported by Sedda et al. (19) and subsequently confirmed by other authors (20–23), who consistently demonstrated excellent results in terms of efficacy, tolerability, and feasibility. More recently, Mirzaei et al. summarized the main technical procedures and available literature (29,30). In our series, we observed an overall efficacy rate of 91.1% after a mean follow-up of 36 mo. It is noteworthy, however, that approximately one third of relapses were marginal, occurring partially outside the field of irradiation (GTV_edge_), particularly in very large lesions (mean surface area, 19 cm^2^; Table 2). This finding highlights the importance of keeping treatment margins as wide as possible when defining the GTV. Nonetheless, this is not always feasible because of the proximity of critical structures such as the eye, mucosa, or nipple. Importantly, even in relapsed cases, treatment often induced a significant reduction in lesion size, thereby facilitating subsequent surgery or alternative therapies (Fig. 2). We identified a statistically significant correlation between relapse rate and lesion size, suggesting that a stricter selection of patients—especially targeting smaller lesions—may further improve efficacy and reduce recurrence. This hypothesis is supported by data from the EPIC Skin study. Baxi et al. (31) reported a partial response rate of 2.8% and a complete response rate of 97.2% after 6 mo in 106 lesions smaller than 8 cm^2^. Cardaci et al. (32) confirmed these results at 12 mo in 185 lesions, with a partial response rate of 5.9% and complete response rate of 94.1%. Similarly, Tietze et al. (33) reported a 95% response rate and 5% relapse rate after 12 mo in 40 lesions of similar size. Applying the same arbitrary 8-cm^2^ cutoff retrospectively to our cohort, we observed excellent outcomes: 2 relapses among 139 lesions at 6 mo (relapse rate, 1.4%; complete response rate, 98.6%) and 5 relapses at 12 mo (relapse rate, 3.5%; complete response rate, 96.5%) (Table 4). Nevertheless, it should be considered that the mean dose delivered in our series at the deepest point of invasion was 25 Gy, significantly lower than the 50 Gy reported in the EPIC Skin study. Moreover, a precise comparison is limited by the incomplete reporting of lesion thickness and surface area in the EPIC cohort (Table 4). A frequent cause of relapse is the underestimation of lesion thickness. In 3 of 8 lesions subsequently retreated with surgery, histopathology revealed a greater thickness than initially expected. In this regard, the use of high-frequency ultrasound may provide additional accuracy in assessing tumor depth (34). Another factor associated with recurrence was the morphoeic BCC subtype, which recurred in 3 of 15 cases (efficacy 80%). Although not statistically significant (likely due to the limited sample size), this finding is consistent with the known biologic behavior of morphoeic BCC, characterized by vertical and early dermal invasion, as opposed to the more superficial horizontal growth of nodular BCC. With respect to safety, the incidence of acute adverse events was relatively low. Most patients (92%) developed CTCAE grades 1–2 toxicity, which was easily manageable and resolved within 3–4 wk after the first follow-up visit. Grade 3 toxicity occurred in 8% of patients, mainly in large-volume lesions, and was characterized by delayed healing (up to 12 wk), requiring local topical support. Only 2 cases (large lesions in the pretibial and breast regions) showed adverse events persisting beyond 12 wk. A significant correlation was observed between adverse event severity and lesion size and volume. Importantly, in the subgroup with long-term follow-up (mean, 66 mo; range, 60–72 mo), no late adverse events attributable to ^188^Re-ERT were observed.

**TABLE 4.** Comparison of Results of Most Relevant Publications in Lesions Smaller Than 8 cm^2^

Study	No. lesions <8 cm^2^	Follow-up at 6 mo CR/RR (%)	Follow-up at 12 mo CR/RR (%)	Mean dose at maximum NMSC invasion	Mean NMSC surface area (cm^2^)	Mean NMSC thickness (mm)
Current study	139	98.6/1.4	96.5/3.5	25 Gy	3.4	1.3
Baxi (31)	106	97.2/2.8	/	50 Gy	NA	NA
Cardaci (32)	185	/	94.1/5.9	50 Gy	NA	NA
Tietze (33)	40	/	95/5	50 Gy	1.2	0.35

CR = complete response; RR = relapse rate; NA = not available.

Based on our experience, the main strengths of ^188^Re-ERT include being a single, fast, and painless application, making it particularly attractive for elderly patients or those with limited therapeutic options; applicability to lesions with complex geometries (e.g., ears, nasal ala) and to large lesions where homogeneous dose delivery by other modalities is difficult; and feasibility in an outpatient setting, with a favorable safety profile and manageable toxicity.

On the other hand, several challenges and limitations must be considered: underestimation of tumor thickness from a single biopsy, which may lead to early recurrence; lesion size and volume, which significantly influence both recurrence risk and toxicity; morphoeic BCC subtype, which is more prone to relapse due to its vertical invasion pattern.

From a technical perspective, the main limitations of ^188^Re-ERT are the physical characteristics of ^188^Re, which limit effective penetration to lesions thicker than approximately 3 mm; the need for dedicated facilities authorized to handle unsealed radioactive sources, which restricts widespread implementation.

Finally, we acknowledge the limitations of our study, particularly its retrospective nature and the advanced age of the enrolled population (mean, 81 y). Approximately one fourth of patients were lost to follow-up because of death from unrelated causes, limiting our ability to evaluate long-term recurrence rates and late toxicity. Nevertheless, it is noteworthy that in the subgroup with extended follow-up (23 patients; mean, 66 mo), no late treatment-related adverse events were observed, and most deaths occurred from unrelated causes.

## CONCLUSION

^188^Re-ERT is a safe, well-tolerated, and effective option for the treatment of NMSC, particularly in elderly patients or those unsuitable for surgery. Our results confirm high response rates, especially in smaller lesions (<8 cm^2^), with manageable toxicity and no late adverse events observed on long-term follow-up. Careful patient selection, accurate assessment of lesion thickness, and consideration of histologic subtype are critical to maximize efficacy and minimize recurrence. Despite technical and logistical limitations, ^188^Re -ERT represents a valuable addition to the therapeutic armamentarium for NMSC in selected patients.

## DISCLOSURE

Paolo Castellucci received consulting fees, travel fees, or honoraria from Novartis, Curium, Recordati, and AstraZeneca. Stefano Fanti reports, outside the submitted work, personal honoraria from Novartis and personal fees from AAA, Amgen, Astellas, Bayer, Debio, GE HealthCare, Immedica, Janssen, SOFIE, and Telix. No other potential conflict of interest relevant to this article was reported.

## Notes

KEY POINTS **QUESTION:** What are the long-term outcomes of patients with NMSC who are difficult to treat or not eligible for surgery and treated with rhenium-based therapy? **PERTINENT FINDINGS:** Long-term follow-up data show that rhenium-based therapy provides effective local control with favorable safety in patients with inoperable NMSC. Recurrence rates remained low over time, and treatment-related adverse events were minimal. **IMPLICATIONS FOR PATIENT CARE:** ^188^Re-ERT represents a valuable nonsurgical treatment option for patients with inoperable NMSC, particularly in elderly or frail populations and for difficult to treat lesions.
